# MR-guidance – a clinical study to evaluate a shuttle- based MR-linac connection to provide MR-guided radiotherapy

**DOI:** 10.1186/1748-717X-9-12

**Published:** 2014-01-09

**Authors:** Tilman Bostel, Nils H Nicolay, Jörg G Grossmann, Angela Mohr, Stefan Delorme, Gernot Echner, Peter Häring, Jürgen Debus, Florian Sterzing

**Affiliations:** 1 Clinical Cooperation Unit Radiation Oncology, German Cancer Research Center (DKFZ), Im Neuenheimer Feld 280, 69120 Heidelberg, Germany; 2 Department of Radiation Oncology, University of Heidelberg, Im Neuenheimer Feld 400, 69120 Heidelberg, Germany; 3 Department of Medical Physics in Radiation Oncology, German Cancer Research Center (DKFZ), Im Neuenheimer Feld 280, 69120 Heidelberg, Germany; 4 Department of Radiology, German Cancer Research Center (DKFZ), Im Neuenheimer Feld 280, 69120 Heidelberg, Germany

**Keywords:** IGRT, MR-guided radiotherapy, Dose reduction, Shuttle

## Abstract

**Background:**

The purpose of this clinical study is to investigate the clinical feasibility and safety of a shuttle-based MR-linac connection to provide MR-guided radiotherapy.

**Methods/Design:**

A total of 40 patients with an indication for a neoadjuvant, adjuvant or definitive radiation treatment will be recruited including tumors of the head and neck region, thorax, upper gastrointestinal tract and pelvic region. All study patients will receive standard therapy, i.e. highly conformal radiation techniques like CT-guided intensity-modulated radiotherapy (IMRT) with or without concomitant chemotherapy or other antitumor medication, and additionally daily short MR scans in treatment position with the same immobilisation equipment used for irradiation for position verification and imaging of the anatomical and functional changes during the course of radiotherapy. For daily position control, skin marks and a stereotactic frame will be used for both imaging modalities. Patient transfer between the MR device and the linear accelerator will be performed with a shuttle system which uses an air-bearing patient platform for both procedures. The daily acquired MR and CT data sets will be digitally registrated, correlated with the planning CT and compared with each other regarding translational and rotational errors. Aim of this clinical study is to establish a shuttle-based approach for realising MR-guided radiotherapy for certain clinical situations. Second objectives are to compare MR-guided radiotherapy with the gold standard of CT image guidance for quality assurance of radiotherapy, to establish an appropiate MR protocol therefore, and to assess the possibility of using MR-based image guidance not only for position verification but also for adaptive strategies in radiotherapy.

**Discussion:**

Compared to CT, MRI might offer the advantage of providing IGRT without delivering an additional radiation dose to the patients and the possibility of optimisation of adaptive therapy strategies due to its superior soft tissue contrast. However, up to now, hybrid MR-linac devices are still under construction and not clinically applicable. For the near future, a shuttle-based approach would be a promising alternative for providing MR-guided radiotherapy, so that the present study was initiated to determine feasibility and safety of such an approach. Besides positioning information, daily MR data under treatment offer the possibility to assess tumor regression and functional parameters, with a potential impact not only on adaptive therapy strategies but also on early assessment of treatment response.

## Background

In the last decade, technological advances in medical imaging significantly affected the specialty of radiation oncology. Today, there is a wide range of applications for modern imaging procedures in the oncological field encompassing diagnosis, follow-up monitoring, prognosis, and assessment of functional/biological parameters of tumor diseases as well as spatial and temporal imaging done prior to each radiation fraction to confirm tumor position (i.e. image-guided radiotherapy, IGRT).

In general, for radiotherapy planning a CT scan is performed with the patient immobilized in treatment position to define the target tissue, to calculate the irradiation plan and to generate reference coordinates for the daily treatment settings. Nowadays, other imaging techniques like MRI including MR spectroscopy, PET or SPECT are frequently used for registration to the CT planning data to enhance tumor delineation and to display functional data about the tumor and surrounding normal tissues [1-5]. In particular, MRI is taken into account for radiation planning more and more frequently due to improved registration algorithms and its excellent soft tissue contrast which allows superior distinction between cancerous and normal tissues [6].

Furthermore, technical advances have enabled the integration of various imaging modalities into the linear accelerator for daily image guidance of radiotherapy, improving the management of inter- and intrafractional variations [7,8]. In particular, the use of CT for daily image guidance marked an important milestone, which enabled improved precision and accuracy in delivering radiation therapy to cancerous tumors while respecting surrounding healthy tissues and organs at risk [9-12]. By deploying IGRT on the basis of CT image guidance, positioning deviations can be corrected instantly and the whole tumor volume can be assessed daily. This is very useful since tumors can change the shape, shrink or expand during therapy and move between treatments due to differences in organ filling, movements while breathing or weight loss of the patient [13-16]. In years past, the problem of localization errors of moving tumors during treatment was compensated by larger planning target volumes (PTVs). This resulted in adjacent normal tissues and critical organs receiving unnecessary doses of radiation during treatment, which, in turn, limited the potential of dose escalation in the primary tumor to improve treatment outcome.

Nowadays, CT-based image guidance is a standard procedure in many radiotherapy units helping the physician to follow tumors as they move and to align the patient to the reference position of the planning CT dataset before each treatment session in case of gross positional misalignments to ensure an accurate adjustment of the external beam. Thus, radiation fields can be confined to the tumor very precisely and radiotherapy can be delivered as planned.

Generally, the use of CT-based IGRT should be considered for the following clinical situations: Cancers with positional variations between the radiation fractions to reduce geographic uncertainties, cancers in which increased dose has been associated with increased local tumor control, tumors with narrow safety margins to dose-limiting healthy tissue structures to achieve a steep decline outside the target (in conjunction with intensity-modulated radiotherapy, IMRT), high conformal radiotherapy of intraabdominal tumors for detection of positional changes due to breathing, peristalsis or different fillings of gastrointestinal tract, and for patients with immobilisation problems due to pain or claustrophobia [11,17].

Furthermore, anatomical changes that require a modification of radiation treatment, e.g. weight loss, strong tumor shrinkage or opening of an atelectasis, can be assessed by IGRT at an early stage in order to take necessary consequences, i.e. re-planning for optimized adaptation to the aimed volume with consideration of the new anatomical situation [16,18]. This is essential for individual dose distributions in highly conformal radiation therapy, with the benefits of dose escalation in tumors and reduced side effects and thus better tolerability.

In addition to the just mentioned benefits, there are also some disadvantages of CT image guidance. Firstly, CT imaging is limited with respect to achievable tissue contrast. Secondly, existing CT solutions for IGRT (cone beam CT, in-room CT) can not provide real time data under treatment, so being unable for instance to follow (irregular) breathing. Thirdly, every CT scan leads to an additional radiation dose to the patient. This is just a small portion of the overall radiation dose that is administered to the patient in the course of radiotherapy, but particularly in the treatment of children and young adults an increased risk for secondary malignancies can not be excluded [19]. However, for the treatment of most (elderly) patients the argument with the additional radiation exposure is just secondary, the main arguments for MRI guided radiotherapy are the much better soft tissue contrast compared to cone beam CT, and furthermore the option for obtaining real time data and tracking of tumors.

The first two points particularly form the rationale for MR-guided radiotherapy to improve delineation of the target volume, thus saving the surrounding healthy tissues and organs at risk, the third point for reliable alternative imaging modalities suitable for image guidance of radiotherapy that do not expose the patients to any additional radiation dose according to the so called ALARA (As Low As Reasonably Achievable) principle. In the years past, several studies reported about the feasibility and accuracy of ultrasound for image guidance of pelvic and upper-abdominal cancers [20-23]. It is a widely used diagnostic tool and quite inexpensive. However, as decisive disadvantages, the ultrasound procedure is prone to interuser variability, requires experienced personell and careful patient selection is necessary due to the fact that good sonographic visibility of upper abdominal cancers strongly depends from the patients’ weight, presence of intestinal gas and preparation of the patient (empty stomach).

As a further alternative to CT, MRI may be considered for image guidance of radiation treatment. Nowadays, MRIs are widely available and excellent at imaging soft tissue structures and therefore an ideal technology for combining with linear accelerators because most cancers occur in soft tissue. Thus, MRI is a promising and suitable imaging method for accurate monitoring of moving tumors and the surrounding tissues in the everyday practice of radiotherapy.

Two study groups from the Netherlands and Canada could demonstrate the proof of concept of an integrated MRI and linear accelerator [24,25]. Up to now, there are only a few prototypes of integrated MRI-linac devices which are still in development and not available for the clinical routine [26]. However, first phantom results of such MR-linac prototypes are very promising [27,28] but it will probably take a few years until current technical problems will be solved and such hybrid devices will be brought to market [29-33].

That is why the concept of a shuttle-based connection between a MRI device and a linear accelerator is a promising alternative to provide MR-guided radiotherapy in the near future which could be used for instance for optimization of adaptive therapy strategies and building first experiences for real-time MRI guided radiotherapy. Due to the fact that MR imaging in the setting of a shuttle-based approach is off line (like existing CT solutions for IGRT), no real time data can be provided.

However, if the uncertainties of the shuttle based approach due to patient transport between the MRI device and linac should remain within certain limits, this off-line solution for MRI guided radiotherapy could be an alternative to CT image guidance in the radiation treatment of children or young adults. Therefore, clinical studies are needed in order to demonstrate the feasibility and safety of such an approach. In the proposed clinical study MR-GUIDANCE (**M**agnetic **R**esonance Tomography-**guid**ed r**a**diotherapy compared to the gold sta**n**dard **c**omput**e**d tomography) patients with tumors located in the head-and-neck, thoracic, upper abdominal and pelvic region will be enrolled receiving standard CT-guided radiotherapy and short additional MRI scans performed in treatment position (including all necessary immobilization devices, treatment supports and stereotactic tools) prior to each radiation fraction. Besides positioning information, MRI-guided radiotherapy offers the possibility to assess tumor regression and functional parameters, with a potential impact not only on adaptive therapy strategies but also on early assessment of treatment response.

## Methods/Design

### Study design

This study is an open, mono-centric, non-randomized, prospective study to evaluate feasibility and safety of a shuttle-based MR-linac connection providing MR-guided radiotherapy for position verification and imaging of anatomical changes during the course of a radiation treatment. Patients eligible for the MR-GUIDANCE trial have to present with the indication for a radiotherapy of the regions head and neck, thorax, upper abdomen and pelvis in the neoadjuvant and adjuvant setting, or as definitive treatment. For validation of MR-based image guidance of radiation treatment, comparison with the gold standard of CT will be performed on a daily basis in the course of radiotherapy. Position correction will be performed for all study patients entirely on the basis of CT imaging.

### Trial organisation

The MR-GUIDANCE trial has been designed by the Departments of Radiation Oncology of the University of Heidelberg in cooperation with the Departments of Radiation Oncology, Diagnostic Radiology, Biostatistics and Medical Physics at the German Cancer Research Center (DKFZ) Heidelberg. The trial is an investigator-initiated trial and is carried out by the Departments of Radiation Oncology and Diagnostic Radiology at the German Cancer Research Center together with the Department of Radiation Oncology at the University Hospital of Heidelberg.

### Coordination

The trial is coordinated by the Departments of Radiation Oncology and Diagnostic Radiology at the German Cancer Research Center (DKFZ) in cooperation with the Department of Radiation Oncology at the University of Heidelberg. The Departments of Radiation Oncology at the German Cancer Research Center and at the University of Heidelberg are responsible for overall trial management, database management, quality assurance, reporting and for the scientific program of all trial related meetings.

### Investigators

Patients will be recruited by the Departments of Radiation Oncology at the University Hospital of Heidelberg and at the German Cancer Research Center (DKFZ) Heidelberg. All investigators are experienced oncologists from the field of radiation oncology.

### Patient selection: inclusion criteria

Patients meeting all of the following criteria will be considered for admission to the trial:

– Written informed consent

– Histologically confirmed tumor of the regions head and neck, thorax, upper abdomen and pelvis

– Karnofsky performance score ≥ 70%

– Age between 18 and 80 years

– Indication for performing a definitive, neoadjuvant or an adjuvant radiation therapy

### Patient selection: exclusion criteria

Patients presenting with any of the following criteria will not be included in the trial:

– Prior radiation therapy within the region planned to be irradiated

– Decompensated secondary diseases of the respiratory organs, cardiovascular system, metabolic system, haematopoietic and coagulation system, kidneys and draining urinary tract

– Contraindications against using MRI (1,5 Tesla) for treatment planning, e.g. cardiac pacemakers and defibrillators, artificial heart valves or cochlea implants

– Weight ≥ 120 kg

– Claustrophobia

– Severe postoperative wound healing problems

– For tumors of the pelvis no hip prostheses

### Study objectives

The main objective is to demonstrate the safety and feasibility of MR-guided radiotherapy by using an air-bearing transfer system for transport between a conventional MRI device and a linac.

The secondary objectives are to

– compare MR-guided radiotherapy with the gold standard of CT image guidance for position control and imaging of anatomical changes during the course of radiotherapy

– analyze which MR sequences are most suitable for MR-guided radiotherapy showing the tumor and surrounding tissues for a representative population of different tumor sites with an acceptable compromise between fast and reliable imaging while allowing sufficient precision for daily position verification.

– evaluate which treatment using supports and immobilization devices are suitable for fixation of the patient on the platform of the shuttle system

– acquire additional diffusion-weighted imaging (DWI) data before the patient receives his or her daily radiation therapy treatment and analyze this data to determine whether quantitative changes of the DWI signal can be an indicator of physiological modifications connected to tumor response; furthermore DWI sequences can be used for localization, e.g. tumor or suspect lymph nodes

– acquire MR perfusion sequences at the beginning, halfway during treatment and at the end of treatment, in order to obtain data about the perfusion patterns of cancerous tissues under therapy, allowing more detailed informations about tumor regression 

– assess the possibility of using MR-based image guidance not only for quality assurance but also for adaptive strategies in radiotherapy

### Study plan

This is a single center study to compare MR-guided radiotherapy with standard CT-guided radiotherapy. Therefore, all eligible patients with tumors of the head-and-neck, thoracic, upper abdominal and pelvic region meeting all of the inclusion criteria and missing all of the exclusion criteria will be informed about the background of this study and will be offered participation. If the patient consented to participation in this study, the patient will be instructed about the study plan and procedures, and the informed consent will be documented in a written form.

All included patients will receive standard therapy, i.e. highly conformal radiation techniques like intensity-modulated radiotherapy (IMRT) with or without concomitant chemotherapy or other antitumor medication, including daily CT image-guidance. The control CT scans prior to every radiation session are performed on a regular basis during the course of precision radiation therapy for verification of patient position and monitoring of anatomical changes. Therefore, no trial specific CT imaging will be acquired and patients will not be exposed to any additional ionizing radiation.

The only burden of the patients in this study is a MR acquisition performed prior to every radiation fraction in treatment position with the same immobilization equipment used for irradiation and the prolonged time needed for the MR scan and the transport from the MR device to the remote linear accelerator (altogether about 15 minutes).

Position control for daily CT imaging will be provided as standard by skin marks for exact target-point localization. For MR imaging, MR-suitable fiducial markers will be used allowing image registration performed under T1- and T2-weighted MR sequences as well as under CT. These multimodality fiducial markers will be placed exactly on the skin marks. Furthermore, as second stereotactic system, an individually customized stereotactic body frame consisting of acrylic glass attached with Gadolinium filled cables will be used for localizing of the target volume. With the contrast agent Gadolinium stereotaxy will be possible not only with MR images but also with CT images due to its high radiopacity. The position correction based on the skin marks will be compared with those based on the reference coordinates of the stereotactic frame. If necessary, translational position correction will be performed by a patient shift based on the contours of soft tissue structures, i.e. particularly on the contours of the target volume, or on the contours of bony structures. Based on this match, the translational positioning error in all three spatial dimensions and the necessary isocenter shift for correction of this error will be documented. The rotational errors will be recorded but not corrected.

The immobilisation of the study patients will be achieved through a vacuum mattress placed within the stereotactic body frame and in case of head and neck cancers with an individually fabricated head mask fitting into the head coil of the MR device.

Depending on the location or motion of the tumor, different MR sequences for position control might be applied, e.g. tumors of the upper abdomen require 4D-MR sequences with high spatial and temporal resolution with low susceptibility for motion artifacts in contrast to more static tumors like those at the base of the skull that primarily require appropriate anatomical imaging. The planned MR protocol includes at least one sequence for imaging of the anatomical structures consisting of a T1- or T2-weighted sequence in axial direction (e.g. T1-SE, T1-FLASH 2D, T2-TSE, T2-HASTE), eventually combined with one balanced sequence (e.g. SSFP), and an additional diffusion-weighted imaging (DWI) sequence to assess whether quantitative changes of DWI signal might be an indicator of early tumor response during therapy. Beside these daily native MR sequences, intravenous application of Gadolinium is planned at the beginning, halfway during treatment and at the end of the treatment, in order to obtain data about the perfusion patterns of cancerous tissues under therapy, allowing more detailed informations about tumor regression. For exact determination which sequence and slice thickness is most suitable for position control and image registration to the planning CT, a sample of applicable MR sequences will be compared in phantom studies.

Patient transfer between the MRI device and the remote linear accelerator will be performed with a shuttle system (Zephyr System, Diacor, USA). This shuttle system is MR-compatible and uses an air-bearing technology that allows the patient to be effortlessly moved by a transfer sled from MR scanner couch to linac without any movement on their part as it utilises the same patient platform with treatment using supports, immobilization devices and stereotactic tools for both procedures. This means that the patient is scanned and treated in the same position, minimising the risk of translational and/or rotational positional changes during transfer between both devices. Thus, maximum use of image based planning data is possible.

**Figure 1:**
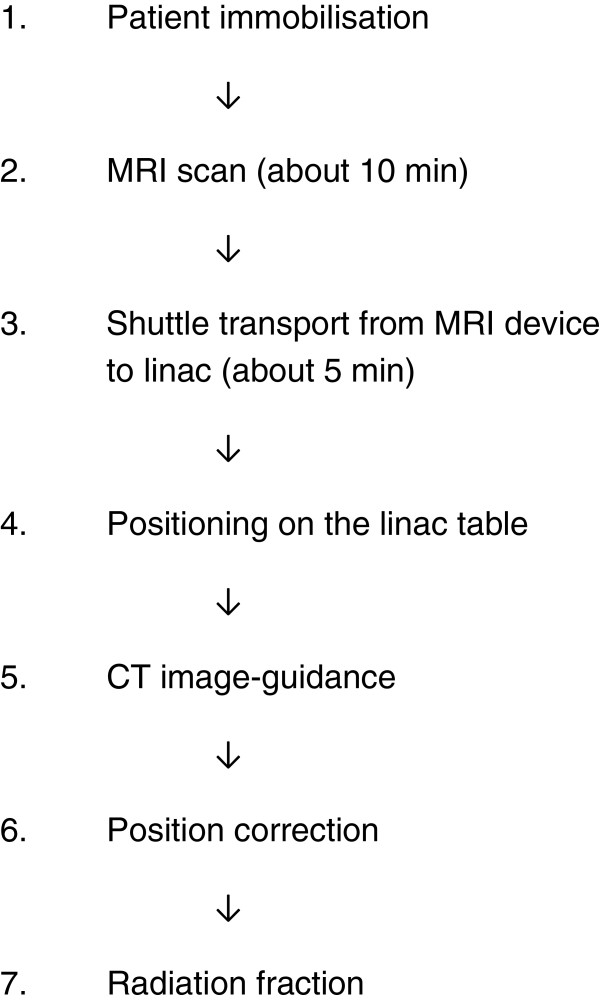
Schematic depiction showing sequence of daily imaging and radiotherapy of study patients.

In the following, acquired MR images will be digitally registrated and correlated with CT planning for calculation of positioning correction vectors. For validation, the correction vectors calculated on the basis of the MR scans will be compared to those calculated on the basis of the CT scans. For safety purposes, position correction will be performed for all study patients entirely on the basis of routinely performed and established CT data (see Figure 1).

### Statistical design

Feasibility and value of pre-radiation MR imaging for planning radiotherapy and correlation with the acquired CT scans for verification of patient position and anatomy will be performed. The positioning accuracy of both imaging modalities will be evaluated regarding translational systematic and random errors and rotational errors in all three spatial dimensions. Therefore, different analysis groups will be evaluated including tumors of the head-and-neck, thoracic, upper abdominal and pelvic region. For optimal calculation and verification, 10 patients per anatomic region will be analyzed, which amounts into a total patient number of 40 patients. For assessment of quantitative change of DWI signal during radiotherapy, correlation with tumor response determined by regular follow-up will be performed.

### Criteria for withdrawal

#### Individual criteria

• Pain in supine position

• Agitated or restless patient

• Claustrophobia

• Discontinuation of study participation by the patient

#### General withdrawal criteria for the study

There are no general withdrawal criteria for the study protocol.

### Prior and concomitant treatments

No prior or concomitant treatment is part of this study. All patients receive standard therapy with radiotherapy alone or in combination with chemotherapy or other antitumor medication in the neoadjuvant, adjuvant setting or as definitive treatment.

### Plan for treatment and care after the trial

After completion of study treatment, no further treatment is planned and patients will be followed up regularly. First follow-up examination will be 6–8 weeks after the end of radiotherapy, further follow-up visits will take place every 3 months for the first 2 years and every 6 months for 3 further years, and after 5 years in annual intervals. They will include clinical and laboratory examination and assessment of acute and late toxicity, and if necessary contrast-enhanced imaging with CT and/or MRI according to the guidelines. The follow-up examinations might be reduced or additional examinations, tests and imaging might be initiated at the discretion of the treating physicians in case of suspicion of local or distant failure. The quantitative changes of DWI signal during radiotherapy will be correlated with tumor response in the follow-up.

### Duration of the study

Patient accrual will start in October 2013 and the end of accrual is planned for March 2015. After completion of this pilot study, all study patients will receive standard follow-up examinations for assessment of therapy response, clinical symptoms, toxicity and quality of life.

The end of this study is defined as the end of the last patient’s therapy. Analysis for the primary and secondary objectives of this study will be performed for each anatomical region alone and finally summarized for all regions together.

### Data collection and management

All clinical and laboratory data and radiotherapy plans will be documented by the investigators or an authorised member of the study team in the patient’s medical record. Important trial documents and the informed consent forms including patients’ consent for trial participation and application of irradiation will be stored and archived according to §13 of the German GCP Regulation and §28c of the German X-Ray Regulation (StrSchV) for at least 30 years after the trial termination.

The Study Center at the Department of Radiation Oncology will be responsible for archiving all relevant data.

### Ethical and legal aspects

The study plan was approved by the Institutional Review Board (IRB)/Independent Ethics Committee (EC) of the Medical Faculty Heidelberg. The trial will be carried out by adhering to local legal and regulatory requirements. The protocol will be conducted according to the Guidelines of Good Clinical Practice (GCP) and the ethical principles described in the Declaration of Helsinki in 2008.

## Discussion

Since 2000, IGRT has been developed and is now been widely introduced into clinical practice. Since this time IGRT has made a remarkable evolution from X-ray to different CT approaches [11]. Existing CT-based IGRT techniques (including both off-board conventional CT and on-board CBCT) are already very effective, but they are limited because it is often very difficult to distinguish tumors from normal tissues in the native contrast with the consequence that there is further potential for planning target margin reduction which is a major concern for adaptive therapy strategies [34]. In contrast to CT, MRI enables a superior distinction between cancerous and normal tissues due to its excellent soft tissue contrast. Thus, currently there are efforts underway to construct a MRI-linac hybrid-machine [26]. In the years past, it was a key challenge bringing the MRI and linac systems together due to interactions between these two devices with compromises in the performance levels [29-31,33,35]. The dutch working group, for example, could solve this problem through active magnetic shielding and smart radiofrequency design in a diagnostic quality closed-bore system [26]. At present, major challenges are for instance the online Monte-Carlo based treatment planning and fast online image registration and tumor tracking [27,36,37].

Furthermore, it can be assumed that such a MR-linac hybrid-machine will be much more expensive than a regular linear accelerator. Therefore, such hybrid-machines will be available to only a few highly specialised centres in the near future. A possible alternative solution to a hybrid-machine could be a shuttle-based connection between a MRI and a linear accelerator to enable MR-guided radiotherapy. The purchase costs for such a shuttle-system would be significantly lower than the hybrid-machine, which makes it available for significantly more institutions.

Moreover, such a shuttle-based solution leaves the freedom to the physicians to use both devices independently of each other. Beyond this study, a shuttle-based approach has the potential for a more flexible use of other different devices, e.g. in the clinical setting of MR-guided brachytherapy, MR-guided radiosurgery, MR-guided particle therapy or PET-imaging after particle therapy.

Aim of the proposed MR-Guidance pilot study is to build first experiences for MRI-guided radiotherapy with the chance to optimize adaptive therapy strategies, and, if successful, to establish a shuttle-based solution for realising MR-guided radiotherapy, and, finally, the integration of MR-guided radiotherapy into the clinical routine especially for the treatment of children and young adults.

## Competing interests

The German Cancer Research Center (DKFZ), Heidelberg, Germany and Diacor, Salt Lake City, USA have a research collaboration.

## Authors’ contributions

FS, TB, SD and JD developed the study concept and planned the trial. TB and FS wrote the study protocol and obtained ethics approval. All authors contributed to drafting the manuscript. GE was responsible for design and construction of a stereotactic frame suitable for CT and MRI based position control prior to each radiation fraction. FS, TB, NHN and AM are responsible for conducting and co-ordination of the trial as well as patient recruitment. All authors read and approved the final manuscript.
